# Effect of Lithium Disilicate Reinforced Liner Treatment on Bond and Fracture Strengths of Bilayered Zirconia All-Ceramic Crown

**DOI:** 10.3390/ma11010077

**Published:** 2018-01-05

**Authors:** Yong-Seok Jang, Hyeong-Rok Noh, Min-Ho Lee, Myung-Jin Lim, Tae-Sung Bae

**Affiliations:** 1 Department of Dental Biomaterials, Institute of Biodegradable Materials, BK21 Plus Program, School of Dentistry, Chonbuk National University, Jeonju 54896, Korea; pooh3180@hotmail.com (Y.-S.J.); dentalmania@hanmail.net (H.-R.N.); mh@jbnu.ac.kr (M.-H.L.); 2 Department of Conservative Dentistry, School of Dentistry, Chonbuk National University, Jeonju 54896, Korea

**Keywords:** bilayered zirconia all-ceramic crown, lithium disilicate reinforced liner, lithium disilicate glass-ceramic veneer, bonding strength, crown fracture strength

## Abstract

This study was performed to evaluate the effect of a lithium-disilicate spray-liner application on both the bond strength between zirconia cores and heat-pressed lithium-disilicate glass-ceramic veneers, and the fracture strength of all-ceramic zirconia crowns. A lithium-disilicate reinforced liner was applied on the surface of a zirconia core and lithium-disilicate glass-ceramic was veneered on zirconia through heat press forming. Microtensile and crown fracture tests were conducted in order to evaluate, respectively, the bonding strength between the zirconia cores and heat pressed lithium-disilicate glass-ceramic veneers, and the fracture strength of bilayered zirconia all-ceramic crowns. The role of lithium-disilicate spray-liner at the interface between zirconia and lithium-disilicate glass-ceramic veneers was investigated through surface and cross-sectional analyses. We confirmed that both the mean bonding strength between the zirconia ceramics and lithium-disilicate glass-ceramic veneers and the fracture strength of the liner-treated groups were significantly higher than those of the untreated groups, which resulted, on the one hand, from the chemical bonding at the interface of the zirconia and lithium-disilicate liner, and, on the other, from the existence of a microgap in the group not treated with liner.

## 1. Introduction

With the increase in patients’ demands for esthetic restoration, a variety of types of ceramic materials have been introduced to improve the esthetics of dental prostheses. Porcelain has several advantages, such as excellent esthetics, chemical durability, and a high compressive strength. Due to these advantages, it has been used in dental clinics as a form of porcelain jacket crown since 1887. However, it has not been widely employed due to fracture problems during mastication. Since then, a metal-ceramic restoration method, where a metal framework with superior mechanical properties reinforces fragile porcelain, has been adopted for the production of esthetic prostheses. However, since opaque prostheses resulting from both the metal framework’s interference of light and the exposure of the margin of metal have been pointed out as the main factor in the degradation of dental esthetics, much attention has been paid to the all-ceramics restoration method, which solely uses ceramic materials with sufficient strength, superior biocompatibility, and penetrability of light [1,2]. Due to their weaker strength and lower esthetics than those of metal-ceramic, several existing all-ceramic prostheses have been restrictively used for the anterior area and not for the posterior area or long bridge. Recent years has seen a new era opening for dental ceramics, as both zirconia ceramics, referred to as ceramic metal, and computer-aided design (CAD)/computer-aided milling (CAM) technology, have been introduced [3].

Zirconia refers to a zirconium (Zr) oxide that has excellent mechanical properties, even being comparable with those of metal. Thus, Garvie et al. claimed that zirconia was a “ceramic steel” and proposed a theoretical model around its superior mechanical properties [4]. Pure zirconia exists as a monoclinic phase at up to 1170 °C, a tetragonal phase from 1170 °C to 2370 °C, and a cubic phase from 2370 °C to 2680 °C, the latter being the melting point as the temperature increases. When the temperature drops after zirconia is calcined, its phase changes from cubic to tetragonal and subsequently, to monoclinic. Among these phase transformations, a tetragonal-to-monoclinic phase transformation shows a highly-rapid martensite-phase transformation, which cannot be suppressed even by rapid cooling [5]. However, if a certain amount of MgO, CaO, and Y_2_O_3_, which are rare-earth metal oxides, is added to pure zirconia sintered at a cubic section, and if this is then followed by heat treatment at a section where the cubic and tetragonal phases coexist, tetragonal crystals are precipitated within the cubic matrix and the transition to the monoclinic phase does not occur, even if the temperature is decreased [6,7]. Thus, tetragonal zirconia polycrystals (TZP), which constitute a metastable state after adding 3 mol % Y_2_O_3_, have widely been used to manufacture zirconia blocks for CAD/CAM processing.

Zirconia has a high refractive index, which is not suitable for dental esthetics, as it can cause color mismatch with adjacent natural teeth. Because of this drawback, when zirconia has been employed in anterior esthetic restoration, porcelain or glass ceramic veneers have been used in the lower part of the zirconia, in traditional all-ceramic crowns for example. Although this two-layer structure is esthetic, there has been a problem with restoration failure due to veneers getting chipped or delaminated unless a strong bond is guaranteed between the two materials [8,9].

There has been much research on the causes of fractures at the interface between the zirconia core and veneer ceramics [10]. It has been shown that the fracture of the zirconia core and veneer ceramics could result from shear stress due to the difference of the thermal expansion coefficients, low wettability of veneer ceramics, shrinkage during firing, and the transformation of zirconia due to heat and stress [11]. Recently, many studies have aimed to improve the mechanical and chemical properties of zirconia surfaces in order to enhance the bonding strength between zirconia and veneer ceramics [12,13,14].

In lately reported studies, lots of surface treatments have been conducted to enhance the adhesion between zirconia and veneering ceramic [15,16,17,18,19,20,21,22]. For instance, the surface roughness of zirconia can be increased by particle abrasion, formation of surface holes by CNC, and laser irradiation using different types of dental lasers and energy intensities [15,16,17,18]. Also, hydrophilic functionality can be offered by using phosphate monomer, and surface energy level can be increased by the cold atmospheric plasma [19]. Chemical bonding can be induced by various kinds of liner treatments and selective infiltration etching (SIE) [15,20,21,22]. Also, the effects of these surface treatments on bond and fracture strengths between zirconia and veneering ceramic have been identified by various mechanical tests, such as shear bond test, microtensile bond strength test, 3 or 4-point bending test, progressive or cyclic load fracture test, and indentation test [23,24,25,26,27,28,29].

This study therefore investigated the effect of a liner treatment on the bonding strength between zirconia cores and lithium-disilicate glass-ceramic veneers, as well as on the fracture strength of bilayered zirconia all-ceramic crown. The null hypothesis that is proposed in the present study is that liner treatment has no effect on either the bonding strength between zirconia cores and veneer ceramics or the fracture strength of bilayered zirconia all-ceramic crown.

## 2. Results

### 2.1. Measurement of the Bonding Strength via a Microtensile Test

**Figure 1:**
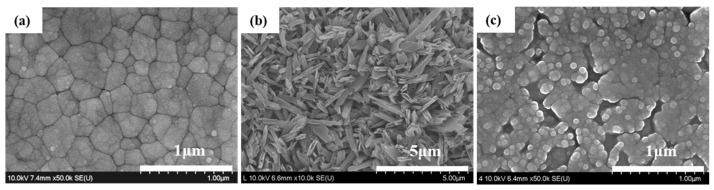
High-resolution field emission scanning electron microscopy (HR FE-SEM) images of (**a**) the sintered surface of 3Y-TZP, and the acid-etched surfaces with a (**b**) 9% hydrogen fluoride (HF) solution for 30 s and (**c**) 5% hydrogen fluoride (HF) solution for 30 min after a spray liner treatment respectively.

Figure 1 shows the results of observations with high-resolution field emission scanning electron microscopy (HR FE-SEM), which investigated the effect of a liner treatment on the evolution of a 3Y-TZP (yttria-stabilized zirconia) surface layer. We observed a typical polycrystalline structure on the surface of 3Y-TZP after maintaining it at 1450 °C for 2 h, as shown in Figure 1a. After applying lithium-disilicate liner to 3Y-TZP and then incubating it at 950 °C for 90 s, the acicular crystal of the lithium disilicate was observed on the surface, acid-etched with a 9% hydrogen fluoride (HF) aqueous solution for 30 s, as shown in Figure 1b. Also, the structural change of zirconia grains was observed at a thin layer on the surface of the specimen, acid-etched with a 5% hydrogen fluoride (HF) aqueous solution for 30 min to remove a liner layer after liner treatment, as shown in Figure 1c.

**Figure 2:**
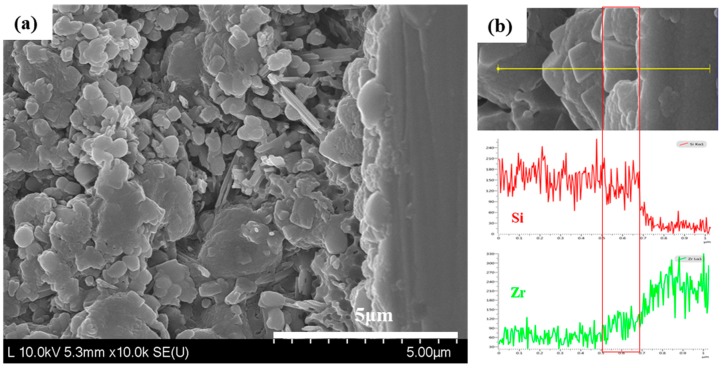
(**a**) HR FE-SEM cross-sectional image and (**b**) energy-dispersive X-ray spectroscopy (EDS) line analysis of the bonding interface after liner treatment on 3Y-TZP.

Figure 2 shows the HR FE-SEM-obtained cross-sectional image of the bonding interface after a liner treatment on 3Y-TZP. The specimen was acid-etched with a 9% HF aqueous solution for 30 s after being cut with a low-speed diamond cutter to observe the bonding interface. We identified that bonding occurred with the formation of a reaction layer on 3Y-TZP’s surface, as shown in Figure 2a. Furthermore, the energy-dispersive X-ray spectroscopy (EDS) analysis results for the reaction layer, as shown in Figure 2b, revealed that there was no significant change in the reaction layer adjacent to the liner, whereas a reduction of the Si peak and elevation of the Zr peak were identified in the reaction layer that was adjacent to the zirconia.

**Figure 3:**
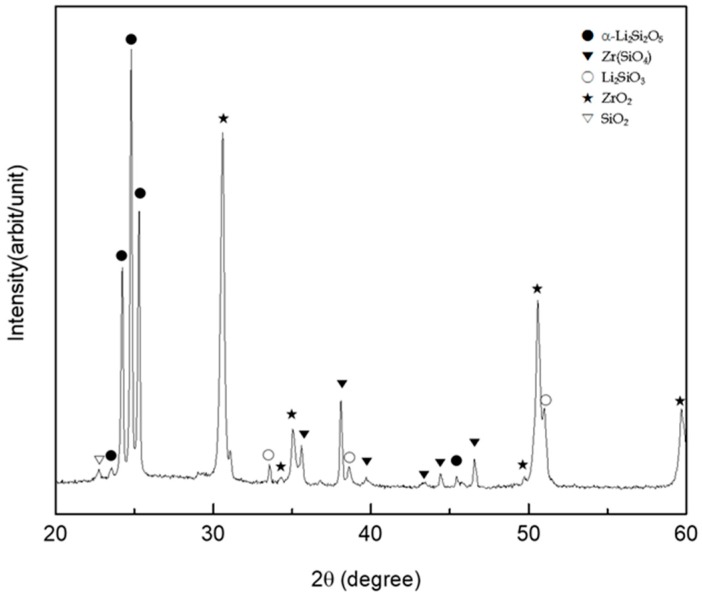
The X-ray diffraction (XRD) pattern on the surface of liner-treated 3Y-TZP.

Figure 3 shows the results of the X-ray analysis on the surface of the liner-treated 3Y-TZP. In addition to α-lithium disilicate (α-Li_2_Si_2_O_5_), zirconia (ZrO_2_), and silica (SiO_2_) peaks, we observed peaks for lithium metasilicate (Li_2_SiO_3_) and zirconium silicate (ZrSiO_4_).

**Figure 4:**
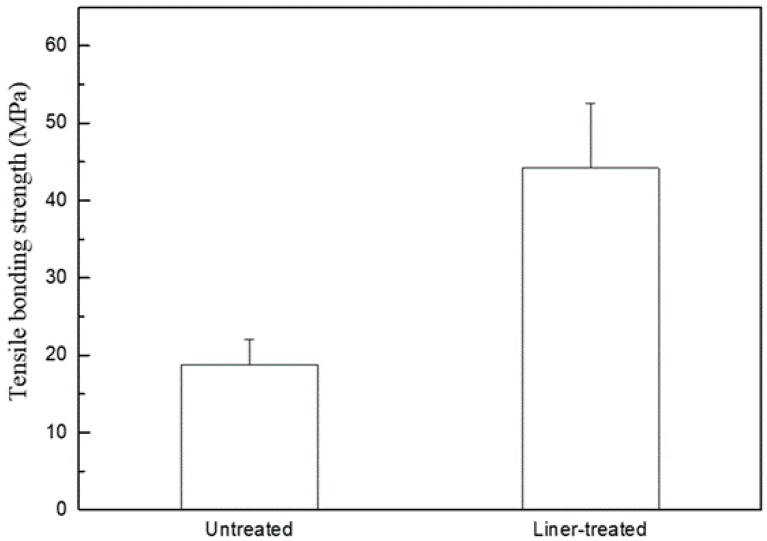
Microtensile bonding strengths after heat press molding with Amber LiSi-POZ of both the untreated and liner-treated 3Y-TZP.

Figure 4 shows the tensile bonding strength after heat press molding with Amber LiSi-POZ of both the untreated and liner-treated 3Y-TZP. The mean tensile bonding strength of the untreated and treated groups was 18.83 MPa and 44.20 MPa, respectively; a statistically significant high bonding strength was revealed in the liner-treated group (*p* < 0.01).

### 2.2. Measurement of the Fracture Strength via a Compression Test

**Figure 5:**
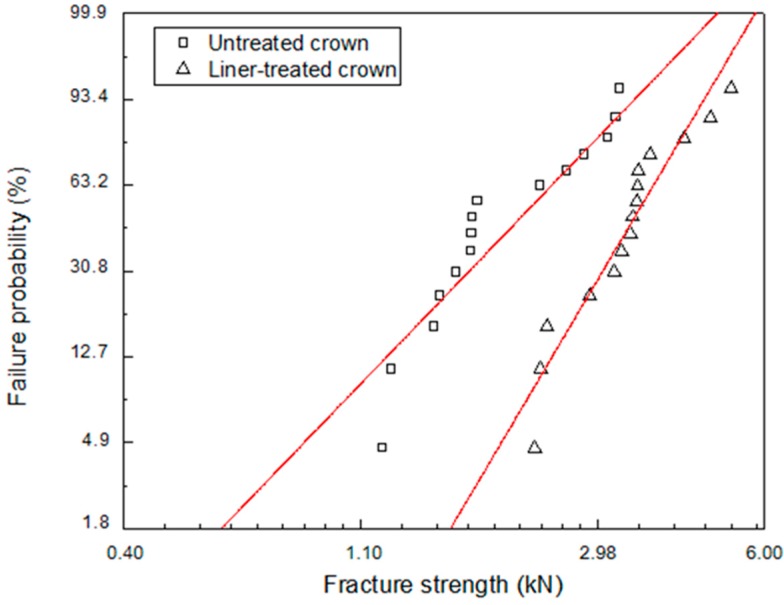
The Weibull plots of the fracture strengths for the untreated and liner-treated bilayered zirconia all-ceramic crowns.

Figure 5 shows the Weibull plots of the fracture strengths for the untreated and liner-treated bilayered zirconia all-ceramic crowns after a crown fracture test, and Table 1 shows the data from a Weibull analysis. The Weibull distribution showed a matched tendency with the single mode (r^2^ > 0.86). The Weibull coefficient of the liner-treated group was higher than that of the untreated group. Additionally, the liner-treated group’s mean fracture strength was, statistically speaking, significantly higher than that of the untreated group (*p* < 0.01).

**Figure 6:**
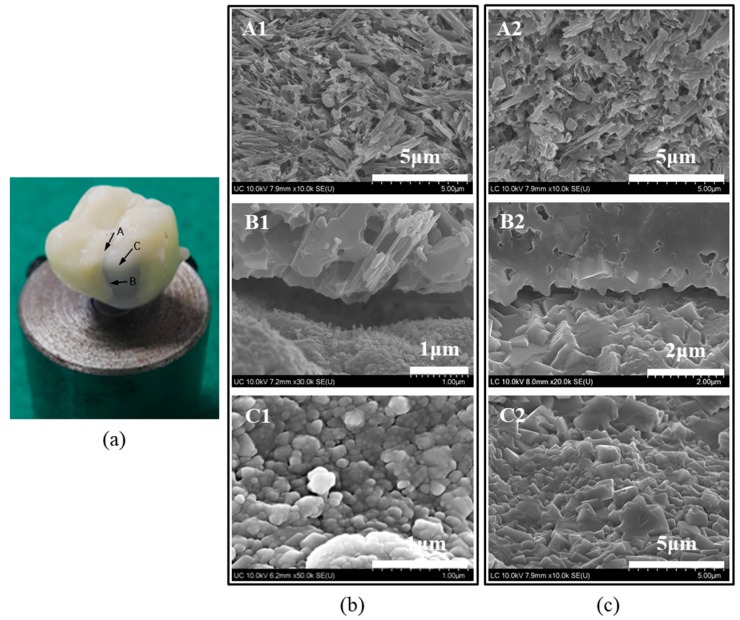
(**a**) Photograph presenting three locations of the fractured bilayered zirconia all-ceramic crown for HR FE-SEM observation; and HR FE-SEM images for (**b**) the fractured surfaces of the untreated and (**c**) liner-treated bilayered zirconia all-ceramic crown s; (A: the veneer’s fracture surface, B: the interface between veneer and core, and C: the core surface).

Figure 6a shows three locations for the HR FE-SEM observation on the fractured bilayered zirconia all-ceramic crown (A: the veneer’s fracture surface, B: the interface between veneer and core, and C: the core surface) after the crown fracture test. Figure 6b,c shows HR FE-SEM images of the fractured surfaces of untreated and liner-treated crowns after the crown fracture test. We observed a typical acicular crystal of lithium disilicate on the veneer fracture surface (A1,A2). In the untreated group, at the interface between the veneer and the 3Y-TZP core, we identified that a small reactive layer was generated on the 3Y-TZP surface (B1), whereas in the liner-treated group, a thick reactive layer formed on the 3Y-TZP surface (B2). The untreated group (C1) showed a similar pattern to that of the sintered surface on the core’s surface, at 1450 °C after milling the block. In the liner-treated group (C2), on the other hand, the reactive layer with the liner completely covered the 3Y-TZP surface.

**Figure 7:**
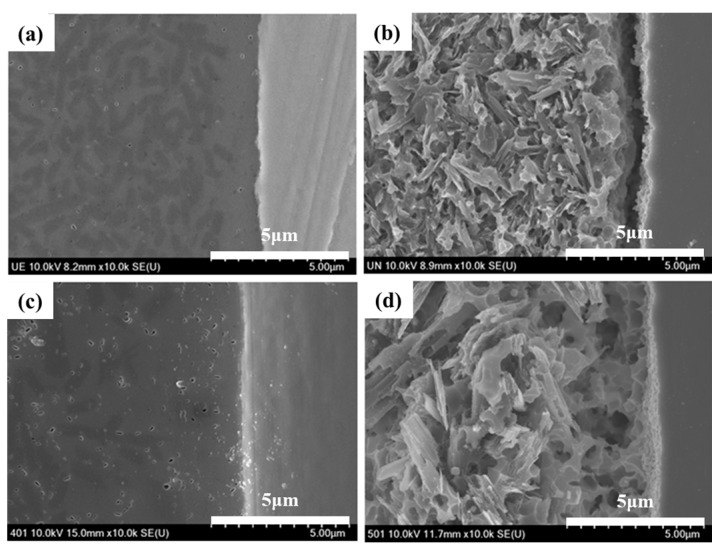
Cross-sectional HR FE-SEM images of the bond interface with and without liner treatment showing the untreated bilayered zirconia all-ceramic crown (**a**) before and (**b**) after acid etching with a 9% HF solution for 30 s; and the liner-treated bilayered zirconia all-ceramic crown (**c**) before and (**d**) after acid etching with a 9% HF solution for 30 s.

Figure 7 shows cross-sectional HR FE-SEM images of the bond interface of the untreated and liner-treated bilayered zirconia all-ceramic crowns before and after acid etching. It was difficult to detect the difference at the bond interface of the untreated and liner-treated all-ceramic zirconia crowns before acid etching, as shown in Figure 7a,b. However, a microgap was observed at the bond interface in the untreated crown, as shown in Figure 7b, but we could confirm a well-combined pattern in the liner-treated crown after acid etching, as shown in Figure 7d.

## 3. Discussion

We conducted this study in order to investigate what the effect of a lithium-disilicate liner treatment applied to a 3Y-TZP zirconia core would be on the bonding strength of lithium-disilicate glass-ceramic veneers and on the fracture strength of an bilayered zirconia all-ceramic crown. It was confirmed that the micro-gap dissipated after the linear treatment due to the chemical reaction at the interface between zirconia core and veneering ceramic during the heat press forming of the veneering ceramics, as shown in Figure 2a and Figure 7d. Thus, the microtensile test was conducted to evaluate the bond strength between the zirconia core and the veneering ceramic. Crown fracture test was also conducted to identify the effect of the microgap on the fracture strength of veneering ceramic when the load is applied on the bilayered zirconia all-ceramic crown consisting of zirconia core and upper veneering ceramic. The results of Figure 4 and Table 1 showed that both the bonding and fracture strengths of the liner-treated group were significantly higher than them of the untreated group, from which we concluded that the present study’s null hypothesis could be rejected.

The strength of the material is an important factor in the chipping of veneer ceramics in zirconia all-ceramic crowns that were made of a zirconia core and upper veneer ceramics. However, both bonding and a matching thermal-expansion coefficient between the core and the veneer hold the highest importance, following the rationale that tensile stress occurring inside veneer ceramic may have a strong influence on chipping. Besides, structural improvements—such as a thickening of the vulnerable areas in veneers where high-tensile stress occurs, a structural design where the core can act as the support, and the prevention of pore-formation inside the sintered body and microgap-formation at the interfaces—are required in order to prevent the formation, via tensile stress, of chipping fractures in veneers [30].

The mismatch of the thermal-expansion coefficient between the core and veneer induces residual tensile stress, which can lead to chipping creation [31]. Since the criteria defining a range for the thermal-expansion coefficient—a range that would be thermally suitable for zirconia all-ceramic restoration—are yet to be clarified, the thermal-expansion coefficient for veneers has been engineered, in metal-ceramic restoration, for instance, to have a slightly smaller value than that of the zirconia core. This aims to induce residual compressive stress on the ceramic-veneer surface. Nonetheless, this has been an issue that generates tensile stress on the surface of zirconia cores [32]. Several researchers have suggested that since residual tensile stress that is caused by the mismatch of the thermal-expansion coefficients may have harmful effects on veneers as well as cores, the thermal-expansion coefficients should be matched for ceramic restoration [33,34].

Since zirconia is chemically stable at a high temperature, its bonding strength with veneer ceramics is low [14]. To overcome this drawback, there have been a number of studies on how to change the structure of zirconia’s surface layer so as to induce mechanical bonding [13,14], and on how liner treatment at the interface induces bonding [12]. The alumina-powder blasting treatment is one of the most frequently used surface-treatment methods for obtaining a mechanical retaining force by changing the surface’s roughness. However, blasting treatment was unable to achieve a high bonding strength due to its limited ability to expand the surface area of polycrystalline zirconia [13]. Since zirconia exhibits excellent stability at a high temperature, it shows chemically inert characteristics for a range of porcelain plastic temperatures. However, when atoms such as Si, Na, K, or Mg are present on the surface, slips, segmentations, and rearrangements of grain boundaries occur. Using this phenomenon, a selective-infiltration etching technique was proposed in order to obtain a microporous structure on the surface [12]. In the present study, we observed a structural change in a thin surface layer of the zirconia in Figure 1c when a lithium-disilicate liner was applied to 3Y-TZP, which was then maintained at 950 °C for 90 s and immersed in a 5% HF aqueous solution for 30 min to remove a layer of liner. We could attribute this structural change in zirconia at the surface layer to the influence of Li and Si, which were included in the liner components, as proposed by Aboushelib et al. [12]. A reaction layer at the interface was generated due to a thermochemical reaction between the zirconia and liner, in which zirconium silicate (ZrSiO_4_) was generated in a layer adjacent to the zirconia, while lithium metasilicate (Li_2_SiO_3_) was generated in a layer adjacent to the lithium-disilicate liner, as confirmed in Figure 2 and Figure 3.

Additionally, the tensile bonding strength of the group treated with the lithium-disilicate reinforced spray-type liner was higher than that of the untreated group, and this was because of the formation of an acicular structure of lithium disilicate in the liner layer during the heat press forming of the veneer ceramics, as confirmed in Figure 1b. Additionally, strong bonding resulted from the formation of zirconium silicate through the reaction of zirconia and silicon dioxide (the liner’s main component) in the surface layer adjacent to the zirconia, whereas a microgap was observed at the interface between the 3Y-TZP zirconia core and veneer in the group that was not treated with liner, as confirmed in Figure 7.

**Figure 8:**
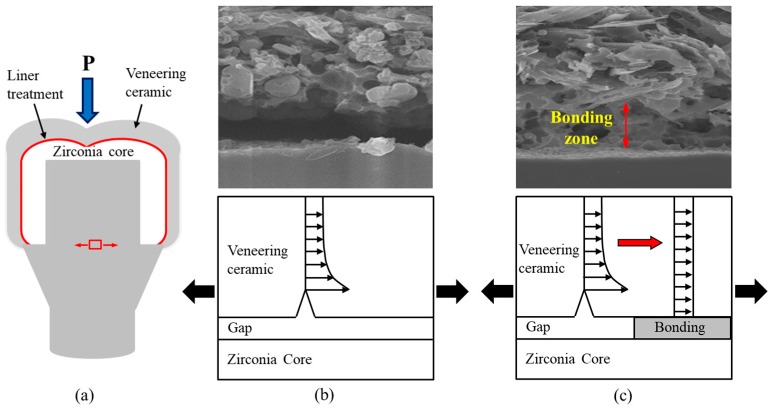
(**a**) An illustration showing the direction of the stress at the edge of bilayered zirconia all-ceramic crown when external force is applied to it; and HR FE-SEM images and figures of the stress distribution at the interface between the core and veneer for (**b**) untreated and (**c**) liner-treated bilayered zirconia all-ceramic crowns.

After the fracture test of the all-ceramic zirconia crown, an interfacial fracture was revealed between the veneer and zirconia core for the untreated all-ceramic zirconia crown. For the liner-treated all-ceramic zirconia crown, a cohesive fracture was revealed in the reaction layer between the liner and core, resulting from strong bonding between the core and veneer materials. The fracture strength of the single crown was significantly higher for the liner-treated crown than for the untreated crown, as was the Weibull coefficient, and this was also confirmed by the figuration of the fracture. This can be explained by the fact that, when external force was applied to the crown, like Figure 8a, for the untreated group, the resistance to fractures dropped due to the stress concentration at the interface, which resulted from the generation of a microgap between the core and veneer, as confirmed in Figure 8b. For the liner-treated group, the resistance to fractures rose due to the stress relaxation at the interface, which resulted from both the elimination of defects in the veneer through the binding of the veneer and liner, and the fact that the core supported the veneer, as confirmed in Figure 8c.

## 4. Materials and Methods

### 4.1. Measurement of Bonding Strength

#### 4.1.1. Preparation of Specimens

To prepare the specimens of the zirconia-sintered body, a calcined 3Y-TZP disc block was prepared for CAD/CAM processing (Zirtooth™ O98FGJ1701, Hass, Gangneung, Korea): it was sintered at 1450 °C for 2 h and then cut so as to measure 12 mm × 12 mm× 7 mm. 50 μm alumina abrasives (Hi-aluminas, Shofu, Japan) were sprayed with a pressure of 3 atm from a distance of 10 mm in order to increase surface roughness in the coupling portion. The specimens were put into an electric furnace and the temperature was increased at a heating rate of 50 °C/min to 1000 °C, and then maintained for 10 min in order to recover a phase-transition layer generated on the surface during the spray-treatment process. Finally, the specimens were then subjected to furnace cooling.

The prepared specimens were randomly divided into the untreated and liner-treated group. For the liner-treated group, a lithium-disilicate reinforced spray-type liner (Hass, Gangneung, Korea), composed of SiO_2_ < 82 wt %, Li_2_O > 10 wt %, P_2_O_5_ < 7 wt %, and other oxides and colorant < 6 wt %, was coated once on the surface of the 3Y-TZP, and the temperature was increased at a heating rate of 60 °C/min to 940 °C and then maintained for 90 s. Following this, a wax pattern that was the same size as the zirconia-sintered body was attached to both the untreated and the liner-treated group, and the investment material (Prime Vest HS, 6-51198-59, Seichong, Seoul, Korea) was used to make a mold. After burning out the wax at 850 °C for 50 min, we conducted heat pressing at 925 °C using lithium-disilicate glass-ceramic ingot (Amber LiSi-POZ, Hass, Gangneung, Korea), as shown in Table 2.

**Figure 9:**
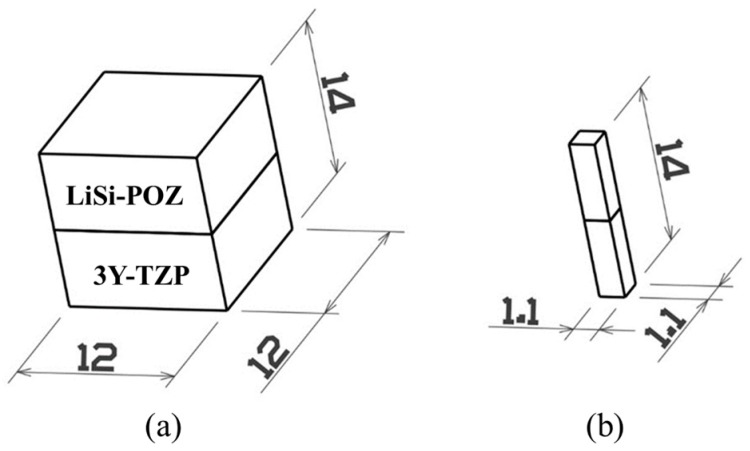
Schematic drawing of (**a**) the coupled block of 3Y-TZP zirconia and LiSi-POZ disilicate glass-ceramic after heat pressing and (**b**) the final specimen prepared for microtensile bond testing.

50 μm glass beads (Rolloblast, Renfert GmbH, Hilzingen, Germany) were blasted at a pressure of 2 atm to remove the investment material that was attached to the specimen’s surface. Then, ultrasonic cleaning was conducted in deionized water for 5 min. The specimens were then fixed to a low-speed diamond cutter and cut into 1.1 mm × 1.1 mm sizes. Four of these cut samples were taken from the center of each specimen. Figure 9 shows schematic drawing to indicate dimensions of the specimens prepared for the microtensile bond testing.

#### 4.1.2. Microtensile Test

A metal holder was attached to both sides of the specimen for microtensile bond testing. The prepared specimens were mounted to a universal testing machine (4201, Instron Co., Norwood, MA, USA), and tensile force was applied at a crosshead speed of 0.5 mm/min in order to measure a load at the moment of an interface fracture. The tensile-bond strength was calculated by dividing a load by a cross-section in the coupling portion. Twelve specimens from each of the untreated and liner-treated groups were used for the microtensile bond testing.

### 4.2. Fracture Test of All-Ceramic Zirconia Crown

#### 4.2.1. Preparation of Specimens

A model of the mandibular first molars of permanent teeth was scanned. Thirty metal abutments were prepared following a customized cutback design. After designing the zirconia coping with a 0.5 mm thickness, a 3Y-TZP disc block (Zirtooth™ O98FGJ1701, Hass, Gangneung, Korea) was processed with the milling machine M1 (Zirkonzahn, South Tyrol, Italy) in order to prepare a total of 30 copings, which were then put into a zirconia sintering furnace (HTC0116, Nabertherm GmbH, Lilienthal, Germany) where the temperature was increased at a heating rate of 3 °C/min up to 1450 °C, and then maintained for two hours.

All of the zirconia copings were treated by blasting with 50 μm alumina abrasives (Hi-aluminas, Shofu, Japan) at a pressure of 3 atm from a distance 10 mm in order to increase surface roughness in the coupling portion. Specimens were divided into the untreated and liner-treated groups, each with 15 randomly allocated specimens: the liner-treated group was obtained by increasing the temperature to 940 °C and maintaining it there for 90 s after applying one coating of spray-type liner.

A crown wax pattern designed and prepared with CAD/CAM was bound to the copings of both the untreated and liner-treated groups, and their molds were made using investment (prime vest HS, BK Giulini, Ludwigshafen, Germany) mixed with water at a ratio of 0.25 and then left at room temperature for 30 min. The molds were heated at 850 °C for 50 min, after which they were coupled to a disilicate glass-ceramic ingot (Amber LiSi-POZ, Hass, Gangneung, Korea) and put into the electric furnace (Programat EP3000/G2, Ivoclar Vivadent, Schaan, Liechtenstein) where they were maintained at 915 °C for 15 min, before being subjected to heat press molding.

**Figure 10:**
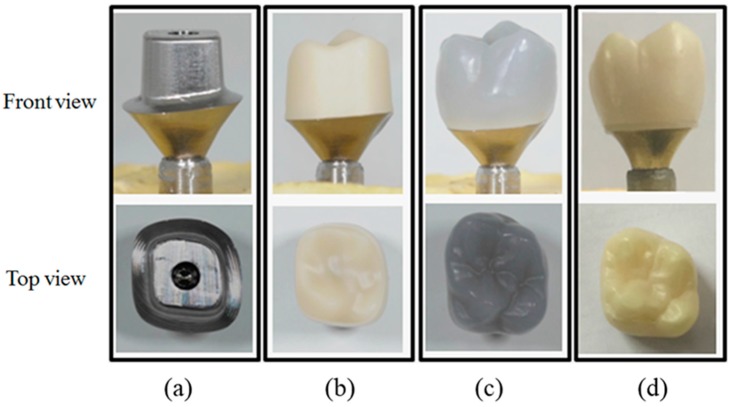
Photograph of the specimen at each stage of the preparation process prior to the fracture test for the all-ceramic zirconia crown; (**a**) custom-milled abutment, (**b**) milled zirconia coping, (**c**) milled wax crown pattern, and (**d**) pressed Amber Lisi-POZ crown.

After molding was completed, the mold was taken out from the electric furnace and then cooled until it became a room temperature. Blasting with 50 μm glass beads was performed at a pressure of 2 atm to remove the investment material attached to the crown’s surface. In order to make it uniform, the surface was then sequentially polished using Carborundum Wheel (GC#22, Sunil Ceramic IND Co., Gyunggido, Korea), Carborundum Point (GC#20, Sunil Ceramic IND Co., Gyunggido, Korea), Silicone Wheel (YoudentTM Classic, Youdent, Shanghai, China), and Rubber Wheel (Dedeco^®^ Classic No. 5001, Dedeco International Inc., Long Eddy, NY, USA). For finishing, we used Kohinoor L (Diamond Polishing Paste No. 516-0001, Renfert GmbH, Hilzingen, Germany). Figure 10 shows the optical images of the specimen at each stage of the preparation process prior to the fracture test for the all-ceramic-zirconia-crown.

#### 4.2.2. Cementation

The metal abutments and the inside of the prepared crown were treated with 32% phosphoric acid gel (ScotchbondTM Universal Etchant, 3M/ESPE, Neuss, Germany) for 30 min and then cleansed with water before being dried. Following this, 10-methacryloyloxydecyl dihydrogen phosphate (MDP)-containing primer (Z-PRIMETM plus, BISCO, Schaumburg, IL, USA) was coated and lightly dried with a drier. We took and evenly mixed a suitable amount of double-polymerized resin cement (Rely-XTM U200, 3M/ESPE, Neuss, Germany), a base agent, and accelerator, which we then filled inside the crown. After this, we applied a compressive force of 49 N using a static-load device to cement the crown to the metal abutment. It was then stored in distilled water at 37 °C for 24 h for an aging treatment.

#### 4.2.3. Crown Fracture Test

**Figure 11:**
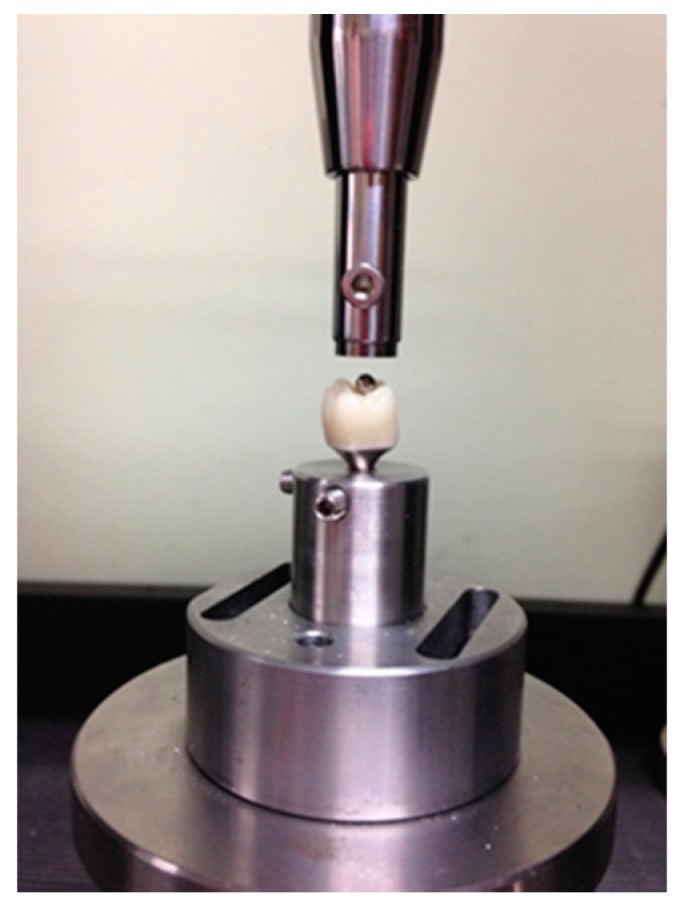
Photograph showing the installation of steel ball and the crown mounted to the universal testing machine for the fracture test of crown.

The prepared crown was fixed vertically on the retainer and then mounted to the universal testing machine (4201, Instron, Canton, MA, USA). A steel ball measuring 3 mm in diameter was placed at the crown’s center and then arranged so as to be placed at the center of the load rod, as shown in Figure 11. Then, a compressive force was applied at a crosshead speed of 0.5 mm/min, and a load was recorded until the moment when a fracture occurred.

### 4.3. Surface Characterization

Using a high-resolution field emission scanning electron microscope (HR FE-SEM, SU8230, Hitachi, Japan), we observed the morphological microstructure of the surface and bond interface after the liner treatment on 3Y-TZP and the fractured surface after the microtensile test, after acid-etching the specimen in a 9% HF aqueous solution for 30 s. Additionally, the surface of the liner-treated 3Y-TZP was acid-etched in a 5% HF aqueous solution for 30 min in order to remove a layer of liner, so as to identify the effect of the liner treatment on the structural change of zirconia grains. We analyzed the concentration change of elements in the reaction layer at the bond interface using energy-dispersive X-ray spectroscopy (EDS, Bruker, Germany). We analyzed the crystal structure of the bond interface after the liner treatment on 3Y-TZP using an X-ray diffractometer (Dmax Ш-A type, Rigaku, Tokyo, Japan).

### 4.4. Statistical Analysis

Data were statistically analyzed using the statistical software SPSS ver12.0 (SPSS Inc., Chicago, IL, USA). A one-way analysis of variance (ANOVA) with the Tukey test was performed in order to compare the bonding strength between the zirconia core and ceramic veneer and the single crown’s fracture strength between the untreated and liner-treated group. We considered a *p*-value of <0.01 as an indication of statistically significant differences.

## 5. Conclusions

The present study aimed to evaluate the effect of liner treatments of 3Y-TZP ceramic cores on the bonding strength of lithium-disilicate glass-ceramic veneers and on the fracture strength of bilayered zirconia all-ceramic crowns. To achieve this, we conducted microtensile bond tests and fracture tests on a bilayered zirconia all-ceramic crown and identified the following results. A chemical bond occurs via the reaction layer in the coupling portion between the zirconia and lithium-disilicate liner. Statistically speaking, both the mean bonding strength between the ceramic zirconia and lithium-disilicate glass-ceramic veneer, and the fracture strength of the liner-treated group were significantly higher than those of the untreated groups. We confirmed that for the untreated group, the resistance to fractures dropped due to the stress concentration at the interface, which resulted from the generation of a microgap between the core and veneer. For the liner-treated group, on the other hand, the resistance to fractures rose due to the stress relaxation at the interface, which resulted from the elimination of microgap between the core and veneer through the binding of the veneer and liner.

In conclusion, given that the chemical bonding occurs at the interface, the application of a lithium-disilicate liner to zirconia and lithium-disilicate glass-ceramic veneering can greatly contribute to improvements in the fracture strength of crowns.

## Figures and Tables

**Table 1:** The Weibull-analysis data of the fracture strengths for the untreated and liner-treated bilayered zirconia all-ceramic crowns.

	Group	Untreated	Liner-Treated
Parameter			
m		2.87	4.68
σ_o_		2.45	3.77
r^2^		0.86	0.91
σ_f(avg)_ ± SD		2.18 ± 0.85	3.45 ± 0.80
N		15	15

m = Weibull modulus; σ_0_ = characteristic strength in kN; r^2^ = Weibull-distribution regression coefficient squared; σ_f(avg)_ = mean fracture strength in N; N = number of samples.

**Table 2:** Firing schedules of the veneering material.

Veneering Materials	ST (°C)	DT (min)	TRI (°C/min)	FT (°C)	V1 (°C)	V2 (°C)	HT (min)
Amber LiSi-POZ	650	1	60	925	650	925	20

ST: starting temperature; DT: drying time; FT: final temperature; TRI: temperature rate increase; V1: temperature of vacuum on; V2: temperature of vacuum off; HT: holding time.
